# Effects of Leucine Supplementation on Athletic Performance, Central Fatigue, and Serum Metabolism in Endurance Athletes: A Randomized Controlled Trial and Targeted Metabolomics Study

**DOI:** 10.3390/metabo16020094

**Published:** 2026-01-27

**Authors:** Tieying Li, Wei Xu, Jun Chen, Zhaobo Kan, Xuemei Sui, Zhiguang Zhao, Qirong Wang

**Affiliations:** 1 Sports Nutrition Center, National Institute of Sports Medicine, Beijing 100029, China; 2 Key Lab of Sports Nutrition, General Administration of Sport of China, Beijing 100029, China; 3 National Testing & Research Center for Sports Nutrition, Ministry of Science and Technology of China, Beijing 100029, China; 4 School of Exercise and Health, Shanghai University of Sport, Shanghai 200438, China; 5 Department of Exercise Science, Arnold School of Public Health, University of South Carolina, Columbia, SC 29208, USA

**Keywords:** leucine, athletes, muscle strength, athletic performance, metabolomics

## Abstract

**Objectives**: We aimed to investigate the effects of 6 weeks of leucine supplementation on athletic performance, central fatigue, and serum metabolome in endurance athletes, and to provide valuable insights into nutritional strategies for endurance athletes. **Methods**: Twenty cross-country skiers were recruited and randomized into 2 groups: the placebo (PLA) group and the leucine (LEU) group. Subjects were given leucine (8.5 g) + sucrose (14 g) or only sucrose (14 g) supplements twice each day from Monday to Saturday for 6 weeks. Test parameters include body composition, aerobic capacity, isokinetic muscle strength, blood biochemistry, and targeted metabolomics. **Results**: After intervention, compared to the PLA group (1) the ankle muscle strength (*p* = 0.01), VO_2_max (*p* = 0.01) and valine in serum (*p* = 0.03) were increased in the LEU group. (2) Targeted metabolomics results showed that the differential metabolites were enriched in the branched chain amino acids (BCAAs) biosynthesis and degradation. (3) The LEU group had a significant increase in α-ketoisovaleric acid (*p* = 0.03), which can reduce the continuous decomposition of BCAAs. **Conclusions**: In conclusion, a six-week intervention of leucine supplementation significantly enhanced ankle muscle strength in endurance athletes, likely through a reduction in BCAA catabolism. Additionally, this combined supplementation strategy demonstrated potential benefits in improving aerobic endurance and may contribute to the attenuation of exercise-induced central fatigue.

## 1. Introduction

In endurance sports, high-level athletic performance depends on the comprehensive enhancement of physiological adaptations induced by long-term, systematic training [1]. Among these adaptations, muscular strength and aerobic metabolic capacity are regarded as two core physiological pillars for endurance athletes [2]. To further unlock their athletic potential, scientific nutritional intervention has become an indispensable component of training programs. Historically, the primary nutritional strategy for endurance training has focused on optimizing carbohydrate (CHO) intake to maintain glycogen stores [3,4]. However, a growing body of evidence suggests that during prolonged, high-intensity exercise, skeletal muscle is not only at risk of glycogen depletion but also faces challenges to its protein balance [5,6]. This imbalance may exacerbate muscle micro-damage and lead to functional decline, thereby becoming a potential bottleneck limiting athletic performance [7]. Against this backdrop, protein supplementation becomes particularly important and has been widely adopted to support recovery and enhance performance [8]. Among its key components, the role of branched-chain amino acids (BCAAs) has attracted increasing attention [9]. Among these, leucine (LEU) has received the most research interest due to its ability to stimulate the initial acute synthetic response and its higher rate of oxidative metabolism compared to the other two BCAAs (isoleucine and valine) [10,11].

The application value of LEU in sports nutrition is primarily based on its physiological functions in protein metabolism [12,13], energy homeostasis [14], and fatigue regulation [15]. Studies have shown that LEU can effectively stimulate muscle protein synthesis and help mitigate exercise-induced muscle breakdown caused by energy imbalance during prolonged exertion [16]. First, as a potent anabolic signal, LEU is crucial for maintaining muscle strength and mass under long-term, high-load training [17]. For example, Negro et al. [18] found that supplementing with LEU immediately after endurance exercise enhanced muscle protein synthesis. However, other researchers have reported that an 8-week LEU supplementation regimen did not significantly improve knee extension strength or muscle cross-sectional area, indicating that the efficacy of LEU in enhancing muscle strength remains controversial [19]. Second, LEU and its metabolites can themselves serve as energy substrates [20]. During exercise, they offer the advantage of bypassing hepatic catabolism and entering the bloodstream directly to fuel skeletal muscle, thereby helping to delay an energy crisis during prolonged activity [21,22]. Furthermore, since BCAAs may modulate central fatigue by competing for blood–brain barrier transporters [23], they have been widely adopted as a nutritional supplement strategy to reduce exercise-induced muscle damage and fatigue. One study found that BCAA supplementation rich in LEU improved subjective fatigue perception during moderate-intensity exercise [24,25]. Additionally, a meta-analysis by Salem et al. suggested that long-term BCAA supplementation (>7 days) could help lower creatine kinase (CK) levels and alleviate delayed-onset muscle soreness [26]. However, a number of studies have also reported that BCAAs show no significant effect in reducing CK levels or mitigating exercise-induced muscle damage and soreness. These findings indicate that conclusions in this field remain inconsistent. Moreover, research specifically involving high-level endurance athletes—particularly studies that use objective measures such as neuroresponse accuracy to quantify central fatigue—is still relatively scarce.

In summary, there remains considerable heterogeneity in the outcomes of LEU supplementation. Furthermore, while most current research focuses on the acute effects of LEU on protein metabolism or its application in resistance exercise, there is still a lack of systematic evidence regarding the direct impact of medium- to long-term LEU supplementation on key athletic performance indicators—such as muscle strength and aerobic metabolism—in high-level endurance athletes. Targeted metabolomics serves as a powerful tool for investigating metabolic changes induced by nutritional interventions. Through quantitative analysis of specific metabolites related to BCAAs catabolism—such as branched-chain keto acids, alanine, and valine—we can gain deeper insights into how LEU regulates athletes’ muscle strength and other physiological adaptations. This study aims to utilize modern mass spectrometry-based metabolomics to explore the effects of six-week continuous LEU supplementation on metabolic changes and athletic performance in endurance athletes, represented by cross-country skiers. It further seeks to examine the correlations among various factors, thereby uncovering the potential biological mechanisms of LEU action at the metabolite level.

## 2. Materials and Methods

### 2.1. Participants

The sample size for this study was determined using G*Power 3.1.9.6. With the Lower Limb Muscle Strength as the primary endpoint and based on previous similar study [27], an independent samples *t*-test estimated that a minimum of 8 participants per group would be required, given an α level of 0.05 (two-tailed) and a statistical power (1-β) of 0.80. To account for an anticipated dropout rate of approximately 20%, this study plans to recruit 10 male cross-country skiers per group.

Twenty national elite male cross-country skiers (Mean ± SD; age: 16.63 ± 1.64 years; height: 174.68 ± 5.65 cm; weight: 63.48 ± 7.18 kg) from the Winter Sports Management Center of Shaanxi Province voluntarily participated in this study. All subjects passed the National second-level athlete certification examination. Considering the subjects’ age, we obtained their coaches’ consent and had each subject sign an informed consent form. Following initial recruitment, we collected baseline information about the athletes. Participants also underwent VO_2_max tests and body composition measurements. Grouping was conducted based on the results of these measurements. There was no significant difference in the baseline indicators (including body composition-related indicators and maximum oxygen uptake) between the two groups.

Exclusion criteria included a history of cerebrovascular diseases, hypertension, diabetes, liver/kidney dysfunction, dairy allergies, gastrointestinal disorders, cardiovascular diseases, and metabolic disorders. All participants signed an informed consent form prior to their involvement in the study. Participant characteristics are shown in Table 1.

### 2.2. Study Design

This study was designed as a single-blind, randomized controlled trial, with 20 participants randomly divided into two groups using a randomization table generated in Microsoft Excel: the PLA group (sucrose, n = 10) and the LEU group (leucine + sucrose, n = 10). Both the leucine supplement and the placebo were contained in identical opaque plastic bottles. Participants in the placebo group consumed a sugar solution that closely matched the leucine supplement in taste, color, and texture. The participants were blinded to the group allocation. One of the 10 subjects randomly assigned to the LEU group was unable to complete the follow-up measures due to injury, and 19 subjects completed the trial. The study was approved by the Ethics Committee of the Sports Medicine Institute, General Administration of Sport of China (Approval No. 202212). The study was prospectively registered with the Chinese Clinical Trial Registry (ChiCTR; registration number: ChiCTR2600116580).

For participants aged under 18: They were fully informed of the study’s purpose, procedures, potential risks and benefits, and signed written informed consent independently. For participants under the age of 18: We obtained written informed consent from their legal guardians and also obtained assent from the minor participants themselves (where appropriate, based on their cognitive ability). Throughout the study, the research team conducted regular monitoring of the physical and psychological status of all participants, and formulated a targeted emergency plan for minor participants, considering their physiological and psychological vulnerability. No study-related adverse events or safety incidents were reported among all participants, including those under the age of 18. Therefore, no ethical or safety issues related to the participants under 18 were identified in this study. All research operations involving minor participants were carried out under the supervision of their legal guardians and the research team, in strict compliance with the ethical norms for clinical research involving human subjects and the relevant regulations for research on minors.

**Figure 1:**
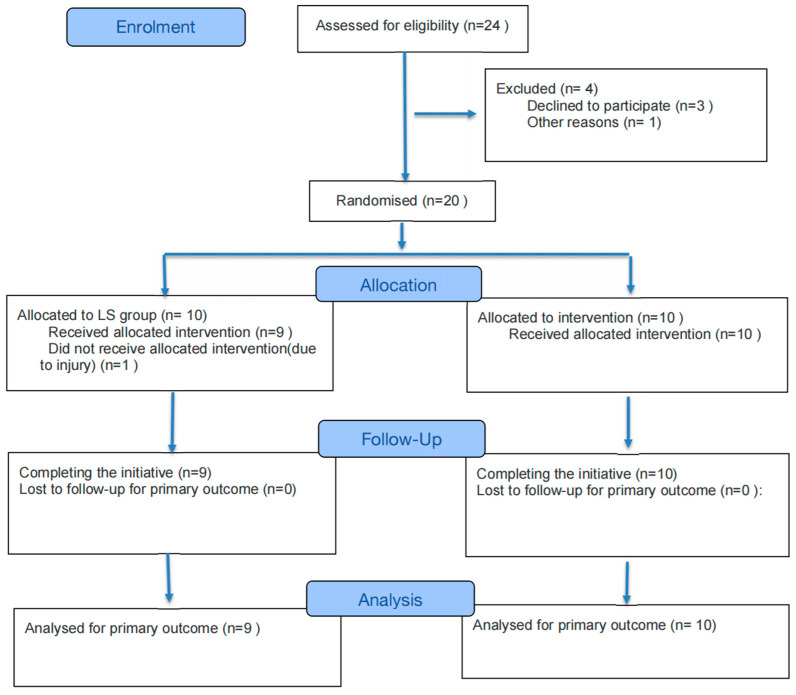
CONSORT 2025 Flow Diagram.

This study strictly adhered to the CONSORT (Consolidated Standards of Reporting Trials) statement to assess the quality of the randomized controlled trial (RCT), including a checklist and a flow diagram. The checklist (see Supplementary File) covers key aspects from trial design, implementation, and analysis to interpretation and reporting. The flow diagram (see Figure 1) describes the progression of participants, from screening and randomization to follow-up and study completion, clearly illustrating the number and flow of subjects at each stage.

### 2.3. Leucine Supplementation Procedure

Participants in the LEU group received leucine (8.5 g, 35.7 kcal) + sucrose (14 g, 56 kcal) or only sucrose (14 g, 56 kcal) supplements twice each day from Monday to Saturday for 6 weeks, while the control group received an equivalent amount of sucrose (28 g/100 mL). The supplementation dose of LEU was primarily based on previous studies [28,29]. The supplementation period lasted for 6 weeks, during which participants followed their regular training schedules and intensity, and adhered to a standardized diet, with the three macronutrients (CHO, fats, and proteins) accounting for approximately 55%, 30%, and 15% of total caloric intake, respectively. Throughout the study, athletes were asked not to engage in weight gain or loss, but to maintain an energy balance, meaning that the energy intake matched the sum of daily metabolism, the thermic effect of food, and physical activity expenditure. Participants ceased training the day before testing, fasted after dinner, with no restrictions on water intake. The measurement of relevant indicators was conducted at the beginning of the trial and after 6 weeks of intervention.

### 2.4. Trial Discontinuation Criteria

A participant will be withdrawn from the trial if they meet any of the following criteria; however, their data collected up to that point may still be included in the statistical analysis upon authorization.

#### 2.4.1. Voluntary Withdrawal of Informed Consent

The participant has the right to withdraw from the trial at any time and for any reason without penalty. No participants in this study requested voluntary withdrawal from the experiment.

#### 2.4.2. Occurrence of a Serious Adverse Event

The investigator determines that a serious adverse event is possibly or definitely related to the study supplement and continued participation would pose an unacceptable risk to the participant. No adverse events occurred during the course of this study.

#### 2.4.3. Onset of a Medical Condition Meeting Withdrawal Criteria

Development of a significant injury or illness (e.g., major infection, fracture, cardiovascular event) during the study that prevents the participant from completing the prescribed training and testing protocols. One participant in this study withdrew from the experiment due to an accidental injury sustained during a training session.

#### 2.4.4. Poor Compliance

Failure to adhere to the supplement regimen (compliance below 80% or above 120%), or failure to complete core tests and sample collections, with no improvement after reminder from the investigator. To ensure optimal adherence, the supplement intervention was administered using a directly observed consumption protocol. A study nutritionist distributed the daily supplement dose to each participant at the designated athlete dining hall. Participants consumed the supplement under the direct supervision of the nutritionist, who verified complete intake. There were no compliance issues in this study.

### 2.5. Standardization and Control of Training, Diet, and Supplementation

#### 2.5.1. Training Control

The Firstbeat Sport Sensor (Firstbeat Analytics, Jyväskylä, Finland) was used to monitor the training load and record the daily energy expenditure. The Firstbeat Sport sensor transmits data online. The Firstbeat heart rate sensor worn by athletes (typically used in conjunction with a heart rate strap) sends real-time heart rate data via Bluetooth to a nearby mobile device (such as a smartphone or tablet). The data received by the Firstbeat Sports app on the mobile device is then uploaded in real time to the Firstbeat Sports cloud server via a network. Administrators can view all athletes’ training data online in real time through the web interface on a computer or the app on a tablet. After training, the system automatically generates an analysis report. During the intervention period, participants wore the Firstbeat device according to the study protocol. Wearing was restricted to daytime periods on planned training days (from 8:00 a.m. to 9:00 p.m., Monday through Saturday).

The training impulse (TRIMP) is a measure used to quantify the training load accumulated during a course. TRIMP considers exercise intensity as calculated using the heart rate reserve (HRR) method and the exercise duration. Training effects (TE) including the aerobic and anaerobic training effects, which were also calculated using HRR. In general, the longer and harder people exercise either aerobic or anaerobic, the higher TE score was expected respectively under the five point-scale in which 0 stands for no effect, 1.0 stands for minor effect, 2.0 stands for maintaining fitness, 3.0 stands for improving fitness, 4.0 stands for highly improving fitness, 5.0 stands for overreaching.

Table 2 lists both the aerobic and anaerobic training effects, total energy expenditure, TRIMP mean daily values, and independent *t*-test *p*-values between groups by the end of each training week.

#### 2.5.2. Diet Control

Participants were advised to maintain their habitual dietary patterns without reducing calorie intake. A nutritionist conducted dietary monitoring twice a week, on Wednesdays and Thursdays, using the 24-h recall method to assess dietary intake. The monitored indicators included total energy intake, protein, fat, and CHO consumption. Portion sizes were estimated using standardized food models to ensure no significant differences in the athletes’ main meals. Additionally, participants were required to keep a detailed daily food intake log, which was reviewed and verified by researchers during the weekly check-ups. Based on the above survey results, we have calculated the energy (PLA: 2525.9 kcal, LEU: 2498.2 kcal) protein (PLA: 86.3 g, LEU: 90.2 g), fat (PLA: 42.7 g, LEU: 39.8 g), and CHO (PLA: 448.7 g, LEU: 445.0 g) consumption of the two groups of subjects. Daily nutritional intake was analyzed using the Dietary Analysis and Management System for Athletes (developed by the National Institute of Sports Medicine, Beijing, China).

#### 2.5.3. Supplementation Control

Participants were explicitly instructed to refrain from taking any medications, stimulants, or nutritional supplements throughout the study.

### 2.6. Outcome Measures

#### 2.6.1. Body Composition

Body composition was measured twice weekly using the InBody 570 bioelectrical impedance analyzer (InBody Co., Seoul, Republic of Korea).

#### 2.6.2. Muscle Strength

(1) Isokinetic Muscle strength

The isokinetic muscle strengths of the ankle and knee joints were quantified using the IsoMed 2000 dynamometer (D. & R. Ferstl GmbH, Hemau, Germany). Before the test, participants completed 3 duplicate practice trials to familiarize themselves with the testing procedure. Before commencing the formal tests, participants completed three repeated practice trials. Subsequently, ankle and knee flexion and extension strength were tested at angular velocities of 60°/s and 180°/s, with five repetitions performed at each speed, and the best result was recorded.

(2) Vertical Jump Test

The vertical jump test was conducted using a Kistler force platform (Kistler, Winterthur, Switzerland). Before the test, the researchers briefly introduced the experimental procedures and requirements to the participants and demonstrated the technical movements of the arm-swing squat jump. Participants performed several practice jumps to familiarize themselves with the movement. The action guidelines are as follows: participants stood upright at the center of the force platform with arms raised vertically and feet shoulder-width apart, then performed an arm-swing squat followed by a vertical jump, ensuring both feet landed fully on the force platform. The force platform was zeroed by the operator, and participants were instructed to “step onto the platform”. This process was repeated for three squat jumps, with the best result recorded.

#### 2.6.3. Aerobic Capacity Assessment (Maximal Oxygen Uptake, VO_2_max)

All participants were equipped with a metabolic system (SCHILLER ERGO AT104, Schiller, Switzerland) and performed a graded intensity exercise test on a treadmill. The experiment protocol was based on the guidelines of Gasparini et al. [30], with appropriate modifications. Participants began with a 2-min warm-up at a speed of 6 km/h and a 0% slope, followed by an initial running speed of 8 km/h for 1.5 min. Subsequently, the speed was gradually increased by 1 km/h every 1.5 min until reaching 11 km/h. At the stage of 11 km/h, an incline was introduced, starting at 1%. The incline increased by 1% every 1.5 min, up to a maximum of 5%, during which participants were required to sprint to exhaustion. The test was automatically terminated when two out of the following three criteria were met: A. The participant’s heart rate exceeded 180 beats per minute; B. The participant was unable to maintain the predetermined intensity even with encouragement; C. VO_2_max was reached, and real-time VO_2_ began to decline. Blood lactate (portable lactate analyzer, Lactate Scout Sport, EKF, Germany) samples were collected immediately after the test and again at 5- and 10-min post-test. Additionally, at these time points, we recorded the participants’ rating of perceived exertion (RPE), relative and absolute VO_2_max, and the respiratory exchange ratio (RER).

#### 2.6.4. Neural Response Accuracy Assessment

The Vienna Test System (VTS; SCHUHFRIED GmbH, Vienna, Austria) is a computerized psychological assessment tool that provides a reliable means of measuring ability and cognitive skills [31]. The VTS includes many sub-tests, and the Cognitron (COG) test was used in this study to evaluate athletes’ concentration and reaction abilities [32]. Testing requirements: Participants were asked to compare abstract figures to a given model and evaluate their complete congruence. After submitting an answer, the next task was automatically presented. Skipping tasks, returning to previous tasks, or correcting responses was not permitted. Regardless of whether the figures were completely congruent, only one response could be provided for each comparison. This study employed the simple test-time scaling (S1) testing method, consisting of 200 items with no time constraints. Variables such as the Mean Correct Rejection Time (in seconds) (Rejection S), Mean Hit Time (in seconds) (Hit S), Total Correct Rejections, and Total Hits (Hits T) were used to assess the precision of neuromotor control, reflecting the accuracy of sustained work. Hits PR represents the percentile rank of Hits T (compared to normative data), indicating a standardized relative performance level. Higher scores on these variables indicate better focus and accuracy during concentrated tasks.

#### 2.6.5. Blood Samples Collection and Measurements

Blood samples were collected into 4.5 mL coagulation-promoting tubes and were centrifuged at 3500 rpm for 15 min. Serum samples were analyzed using a Mindray BS420 Biochemical Analyzer (Shenzhen Mindray Scientific Co., Ltd., Shenzhen, China) to detect total cholesterol (T-CHO), triglycerides (TG), high-density lipoprotein (HDL-C), and low-density lipoprotein (LDL-C), Creatine Kinase (CK), Uric acid (UA), Creatinine (Cr), Blood urea nitrogen (BUN), Total Bilirubin (TBIL), Direct Bilirubin (DBIL), Indirect Bilirubin (IBIL). Cortisol (C) and Testosterone (T) levels were quantified using a Beckman Coulter DxI 800 immunochemiluminescence analyzer (Beckman Coulter, Inc., Brea, CA, USA). Serum concentrations of Glutathione (GSH), Malondialdehyde (MDA), and Superoxide Dismutase (SOD) were determined via microplate array kits following the manufacturer’s protocols via microplate reader (PerkinElmer, Inc., Waltham, MA, USA).

#### 2.6.6. Serum Amino Acid Content

A volume of 400 μL of acetonitrile was added to 200 μL of serum samples. The samples were then vortexed, mixed, and left to stand at room temperature for 10 min. Subsequently, the samples were centrifuged at 12,000 rpm for 10 min. The resultant upper layer was then blown dry with nitrogen at 40 °C. Finally, the samples were re-dissolved with 100 μL of deionized water for online detection. The chromatographic column (Agilent Infinity Lab (Waldbronn, Germany) Poroshell 120 HILIC-Z, 2.1 mm × 150 mm, 2.7 μm) was operated at 25 °C. The mobile phase A was 20 mmol/L aqueous ammonium formate (pH = 3), and the mobile phase B was a 20 mmol/L aqueous ammonium formate acetonitrile/water (9:1) solution (pH = 3). The mobile phase flow rate was set at 0.5 mL/min, and the sample injection volume was 2 μL. The linear elution gradient of the mobile phase was as follows: from 0 to 11.5 min, mobile phase B decreased from 100% to 70%; from 11.5 to 15 min, mobile phase B increased from 70% to 100%. The mass spectrometry detection conditions were as follows: The curtain gas was set to 20, the collision gas was set to Medium, the Ion Spray Voltage (IS) was set to 5.5 kV, the Temperature (TEM) was set to 330 °C, the Ion Source Gas 1 (GS1) was set to 20, and the Ion Source Gas 2 (GS2) was set to 0.

#### 2.6.7. Metabolomic Analysis of Blood

Fasting blood collection was scheduled for Monday mornings of the 1st and 7th weeks. Blood samples were collected into 1 mL EDTA-containing centrifuge tubes for plasma. The analytical instrument for this experiment was an LC-MS QTRAP 6500+ (SCIEX). The samples were analyzed in both positive and negative ion modes using a spray voltage of 4.5 kV and a capillary temperature of 350 °C. The mass scanning range was set at 50–1500 *m*/*z*. The nitrogen sheath gas and nitrogen auxiliary gas were set at flow rates of 30 L/min and 10 L/min, respectively. The HPLC-MS system was run in binary gradient mode. The mobile phase consisted of (A) a 0.1% formic acid aqueous solution and (B) a mixed acetonitrile–isopropanol solution. The gradient was set as follows: 0–1 min (5% B), 1–5 min (5–30% B), 5–9 min (30–50% B), 9–12 min (50–78% B), 12–15 min (78–95% B), 15–16 min (95–100% B), 16–18 min (100% B), 18–18.1 min (100–5% B), and 18.1–20 min (5% B). The flow rate was set to 0.2 mL/min. The pooled QC sample was injected five times at the beginning to ensure system equilibrium, and then, it was injected every five samples during plasma sample detection to further monitor system stability. A Waters’ BEH C18 column (2.1 mm × 10 cm, 1.7 μm, Waters) was used for all the analyses. The scaling method used in the PCA analysis was pareto correction, and the transformation method used was the log correction. Two hundred repetitions were performed to avoid model over-fitting, and the VIP of each metabolite was obtained. Since univariate analysis is the simplest and most commonly used method for analyzing differential metabolites between two groups, the univariate analyses were also performed with fold change (FC) analysis and *t*-test to obtain FC value and *p* value using the R package metaX (version 1.4.2), respectively. For differential metabolites, metabolic pathway enrichment analysis was carried out based on the KEGG database, and metabolic pathways with *p* < 0.05 were significantly enriched by differential metabolites.

### 2.7. Statistical Analysis

Statistical analyses were performed using Statistical Package for Social Sciences (SPSS v. 27, IBM Statistics, Armonk, NY, USA). Data were presented as mean ± standard deviation (Mean ± SD), and the significance level was fixed at 0.05. The normality of the data distribution was tested using the Shapiro–Wilk method. Two-way ANOVA (time and supplementation) was used to compare the differences between groups. If there was an interaction effect between the time-point and the LEU supplementation, we continued with a simple main effect analysis. If there was no interaction effect, we evaluated the intervention effects of participants between groups using the independent *t*-test. Spearman’s rank correlation coefficient was used to assess the correlations between the most significantly altered metabolites among the groups and indicators of exercise performance and central fatigue (Appendix A.5). In this study, all analyses were conducted using complete cases. For any analysis involving variables with missing values, we employed listwise deletion, whereby an entire case was excluded from the specific calculation if it had any missing data on the relevant variables. This approach ensures the integrity of the analytical dataset.

## 3. Results

### 3.1. Primary Outcomes

#### 3.1.1. Muscle Strength

The strength data are presented in Table 3. Compared to pre-intervention levels, Vertical Jump performance was significantly increased in the LEU group after 6 weeks (*p* < 0.05). Compared to the control group, although not significant, the LEU group showed greater improvement in ankle plantarflexion strength at 180°/s after 6 weeks of intervention (*p* > 0.05), but there was a significant increased peak torque of ankle plantarflexion at 60°/s (*p* < 0.05).

#### 3.1.2. Serum Branched-Chain Amino Acid Content

After 6 weeks of intervention, the levels of BCAA (Leucine, Isoleucine and Valine) in the serum of both the PLA and LEU groups were significantly increased, and at the 6th week, the valine level in the LEU group was significantly higher than that in the PLA group. Details are provided in Table 4.

#### 3.1.3. Targeted Metabolomics

##### Overview of Targeted Metabolomic Analysis

**Figure 2:**
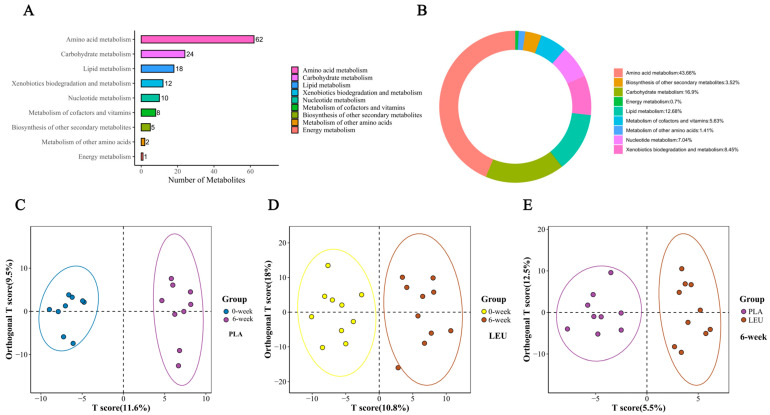
Metabolomics overview. Note: (**A**) and (**B**) display the identified metabolites, with counts categorized according to their final Class. (**A**) The vertical axis represents the class to which a metabolite belongs, and the horizontal axis indicates the number of metabolites. (**B**) Different colors in the chart correspond to distinct metabolite classification categories; the percentage shown reflects the proportion of each category relative to the total number of classified metabolites. (**C**–**E**) present the results of orthogonal partial least squares discriminant analysis (OPLS-DA) for the following paired group comparisons: PLA (0-week/6-week), LEU (0-week/6-week), and 6-week (LEU/PLA). In these plots, the horizontal axis represents the predicted principal component score of the first principal component, and the vertical axis shows the orthogonal principal component score. Each point corresponds to a sample, with different colors indicating distinct sample groups; the ellipses denote 95% confidence intervals.

As shown in Figure 2A,B, a total of 62 amino acid metabolism-related metabolites were detected by targeted metabolomics, accounting for 43.66% of the detected metabolites. Figure 2C–E shows the intergroup OPLS-DA results, from which it can be seen that there are significant differences between the two groups. The raw metabolomics data have been uploaded to the official MetaboLights website.

##### Metabolic Pathway Enrichment Analysis of Differential Metabolites

**Figure 3:**
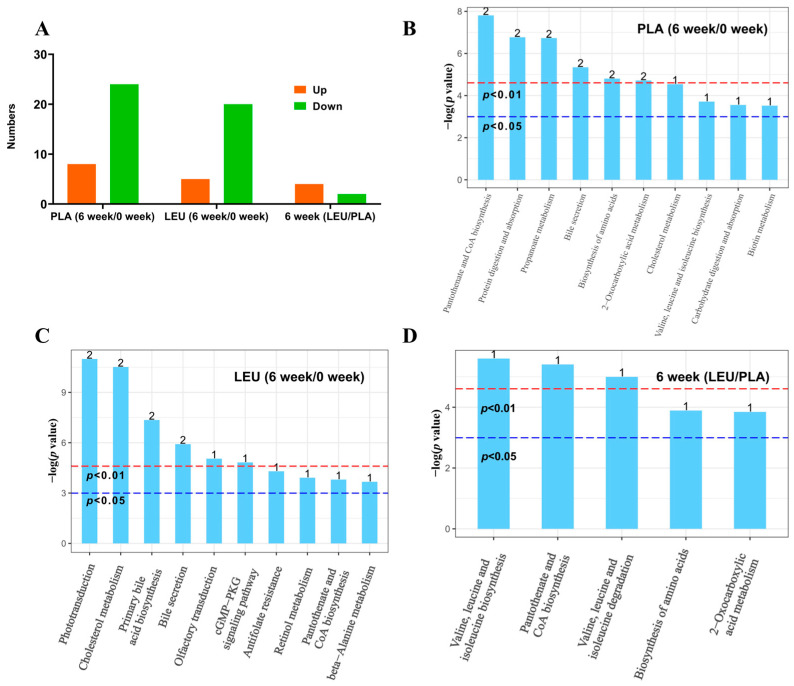
Metabolic pathway enrichment analysis of differential metabolites. Note: (**A**) Number of metabolites whose concentrations differed significantly between groups. (**B**–**D**) shows the results of metabolic pathway enrichment analysis based on differential metabolites between two groups, performed using the KEGG database. Metabolic pathways with a –log (*p*-value) above the red reference line in the figure have *p* < 0.01, while those above the blue reference line have *p* < 0.05. The numbers on the bars indicate the count of differential metabolites mapped to each pathway. (**B**) PLA (0-week/6-week). (**C**) LEU (0-week/6-week). (**D**) 6-week (LEU/PLA).

As shown in Figure 3A, after 6 weeks of intervention, the PLA group had 8 significantly up-regulated metabolites and 24 significantly down-regulated metabolites. The LEU group had 5 significantly up-regulated metabolites and 20 significantly down-regulated metabolites. There were 4 significantly up-regulated metabolites and 2 significantly down-regulated metabolites between the LEU group and the PLA group. As shown in Figure 3B–D, after 6 weeks of intervention, the differential metabolites were enriched in the Pantothenate and CoA biosynthesis, the Protein digestion and absorption and the Propanoate metabolism pathway in the PLA group. The differential metabolites were enriched in the Phototransduction, the Cholesterol metabolism and the Primary bile acid biosynthesis pathway in the LEU group. The differential metabolites were enriched in the valine, leucine and isoleucine biosynthesis, the Pantothenate and CoA biosynthesis and the valine, leucine and isoleucine degradation pathway between the PLA and LEU group.

##### Differential Metabolites Between the LEU and *PLA Group*

After the 6-week intervention, compared with the PLA group, there were 4 metabolites significantly up-regulated in the LEU group, including β-Hyodeoxycholic acid, 23-Norcholic acid, α-ketoisovaleric acid and Glycylleucine, while Desaminotyrosine and β-(m-Hydroxyphenyl) hydracrylic acid were significantly down-regulated metabolites in the LEU group. Details are provided in Table 5.

### 3.2. Secondary Outcomes

#### 3.2.1. Body Composition

There was no significant change in the body composition of the participants in either the PLA or LEU group over 6 weeks (Table 6).

#### 3.2.2. Aerobic Capacity

As shown in Table 7, compared to pre-intervention levels, RER levels were significantly improved in both the PLA and LEU groups after 6 weeks (*p* < 0.05), but there was no significant difference between the two groups. Furthermore, after 6 weeks of intervention, the VO_2_max value was significantly increased in the LEU group compared to the PLA group (*p* < 0.05).

#### 3.2.3. Neural Response Accuracy

The results of the fatigue-related indicators are presented in Table 8. After 6 weeks of intervention, the Hits T and Hits PR values were significantly increased in the LEU group compared to the PLA group (*p* < 0.05).

## 4. Discussion

### 4.1. LEU Supplementation Improves Ankle Strength via Reducing BCAA Catabolism

LEU is regarded as a key amino acid for enhancing athletic performance and muscle function. During physical activity in particular, it is recognized for its central role in stimulating muscle protein synthesis, thereby contributing to increased muscle strength [33]. After a 6-week intervention with daily supplementation of 17 g LEU to skiers, we found no significant difference in vertical jump height between the two groups. However, the ankle muscle strength at 60°/s significantly increased in the LEU group (Table 4). This may due to cross-country skiing primarily relies on type I slow-twitch muscle fibers, which perform better at low speeds and high-power output. On the other hand, improvements in explosive power are more dependent on neuromuscular system adaptations, such as more efficient recruitment of fast-twitch muscle fibers and quicker reaction times. However, LEU does not participate in or directly affect these neural adaptation processes [34,35]. This finding is similar to the results of Howatson et al. [36], who reported that daily supplementation with 20 g of LEU-rich BCAA in facilitated recovery of lower limb strength in the participants, although there was no significant change in vertical jump height. Similarly, Kirby et al. [37] investigated the effects of daily supplementation with 250 mg/kg of LEU on strength in young men. Although no differences in jump height were detected, LEU somewhat inhibited the decline in peak force after exercise. In summary, it is generally clear that LEU supplementation can enhance muscular strength. However, there are still opinions suggesting that for young, healthy individuals who already consume adequate dietary protein (>1.6 g/kg/day), LEU supplementation may not provide additional benefits in terms of increasing muscle strength or mass [38]. Moreover, high doses of LEU can lead to elevated blood ammonia levels and other metabolic issues. Therefore, further studies are needed to refine research, including varying doses, timing of intake, and the effects on athletes in different sports.

To further explore the mechanisms by which LEU supplementation enhances muscle strength, we performed targeted metabolomics analysis. The results showed that after six weeks of nutritional intervention, blood β-alanine levels increased significantly in both the placebo (PLA) and LEU groups (Table 5). This may be explained by the fact that the oxidative metabolism of LEU may involve the consumption of other cofactors, indirectly influencing the carnosine synthesis pathway in which β-alanine participates. Interestingly, metabolomic analysis also revealed that after six weeks of nutritional intervention, the LEU group exhibited a significant elevation in blood levels of α-ketoisovaleric acid—a metabolic intermediate in valine synthesis from pyruvate—compared to the PLA group (Table 5). Consistent with this, valine levels were also significantly higher in the LEU group (Table A4, Appendix A). β-hyodeoxycholic acid can promote intestinal fat breakdown and the absorption of fat-soluble vitamins.

Interestingly, following a 6-week intervention, a significant increase in blood levels of BCAAs was observed in both groups. One potential explanation for this observation is that the nutritional consultant meticulously monitored the dietary intake of study participants during all three meals over the course of the intervention period, thereby prompting heightened awareness regarding protein consumption. It is plausible that this increase may also be an effect of the 6-week exercise training. Moreover, metabolomic results also showed that after six weeks of nutritional intervention, compared to the PLA group, the LEU group had a significant increase in the blood levels of the metabolite α-ketoisovaleric acid which is a metabolic intermediate in the synthesis of valine from pyruvate (Table 5), along with a significant increase in valine levels (Table 4). This may be due to the supplemented LEU competitively binding to key enzymes shared in the branched-chain amino acid metabolic pathway, such as branched-chain amino acid aminotransferase (BCAT) and the branched-chain α-keto acid dehydrogenase complex (BCKDH), thereby inhibiting the normal catabolism of valine [39]. In other words, additional LEU supplementation may preserve the valine from its utilization, evidenced by higher blood valine levels. However, it is worth noting that BCKD, the core rate-limiting enzyme regulating BCAA catabolism in the body, has been shown in some studies to be activated by α-ketoisovaleric acid—a finding that appears contradictory to the hypothesis proposed in our study [40]. Given that the metabolite association data presented here do not establish causality, and since the proposed mechanistic hypothesis is primarily inferred from known metabolic pathways and well-established biochemical principles, further high-quality research will be needed to explore and analyze these relationships in greater depth.

### 4.2. LEU Supplementation Enhances Aerobic Capacity

It is widely recognized that VO_2_max is a key indicator for assessing aerobic endurance. It is influenced by multiple factors, including cardiopulmonary function, the oxygen-carrying capacity of the blood, and the ability of muscles to utilize oxygen. Although some believe that LEU supplementation alone is insufficient to improve VO_2_max [41], our study found that compared to the PLA group, the LEU group showed a significant increase in VO_2_max (Table 7). This suggests that LEU may contribute to enhancing the aerobic capacity of endurance athletes to some extent. Similarly, Matsumoto et al. [42] found that after administering BCAA beverages or isoenergetic placebo beverages to participants, the VO_2_max in the BCAA group was significantly higher than that in the placebo group. This effect may involve multiple mechanisms related to metabolic pathways and physiological adaptations. For example, studies indicate that BCAAs can optimize energy metabolism by activating key metabolic pathways within muscle tissue. An important aspect of this process is the promotion of fatty acid mobilization and utilization, which helps to preserve muscle glycogen reserves and thereby contributes to improved endurance performance [43]. Metabolomic results showed that after six weeks of nutritional intervention, compared to the PLA group, the LEU group had a significant increase in the blood levels of two bile acids, β-Hyodeoxycholic acid and 23-Norcholic acid (Table 5). β-Hyodeoxycholic acid can promote intestinal fat breakdown and the absorption of fat-soluble vitamins while reducing blood cholesterol and triglyceride levels (although no significant changes were observed in our data, see Table A1, Appendix A). The observed increase in muscle mass and enhanced lipid metabolism may have contributed to the improvement in VO_2_max. However, the direct impact of LEU on VO_2_max remains at an exploratory stage, with no conclusive evidence available to date. Therefore, while BCAA supplementation appears to be an effective strategy for improving endurance performance, the underlying mechanisms continue to be a subject of debate.

### 4.3. LEU Supplementation Improves Neural Response Accuracy

The central fatigue hypothesis proposes that prolonged exercise or cognitive tasks lead to decreased efficiency in neural information processing within the brain, manifested as reduced response accuracy and increased decision-making errors [44]. In recent years, numerous studies have employed the COG test to evaluate athletes’ cognitive abilities, reporting that the VTS can objectively assess attention and response accuracy, with results linked to facilitated fatigue recovery [32,45,46,47]. In this study, the test results revealed that after 6 weeks of supplementation, the Hits T and Hits PR of the subjects in the LEU group were significantly improved (Table 8), indicating that their cognitive function was significantly better than that of those in the PLA group, suggesting that LEU plays a certain role in improving exercise-induced central fatigue. Considering that blood biochemical markers also serve as important indicators of fatigue, we measured several relevant parameters (including T, C, CK, SOD, and GSH). However, this study failed to find significant between-group differences regarding fatigue-associated blood biochemical markers (Table A1 and Table A2, Appendix A). Although some scholars have reported that the activities of various antioxidant enzymes (such as SOD and GSH) in animal serum and liver and muscle tissues are increased after LEU supplementation [48], these results were not confirmed in the present human studies. Therefore, LEU supplementation may not be an effective means to promote fatigue recovery by improving the body’s anti-oxidation levels, and further investigation is needed in the future. It is worth noting that assessing fatigue through computerized tests measuring neurocognitive accuracy appears more suitable for athletic populations than blood biochemical markers. This is because cognitive tests directly evaluate core capacities such as attention, reaction speed, decision-making accuracy, and hand-eye coordination—precisely the abilities critical to success in many sports (e.g., ball games, fencing). Fatigue often impairs these higher-order neural functions first.

### 4.4. Limitations

The present study has several limitations. First, the sample size was relatively small (N = 20), constrained by the limited availability of high-level athletes. Future research should aim to recruit larger cohorts to improve statistical power. Second, the experimental design compared only a combined LEU+CHO group with a CHO-only placebo group, lacking a standalone LEU group. A multi-arm design in future studies would help distinguish the independent and synergistic effects of LEU and CHO. Third, the subjects of this study are a specific group of adolescent cross-country skiers, which may limit the applicability of the conclusions. Therefore, it is recommended that future research conduct nutrition intervention studies targeting other typical endurance sports. Finally, while this study utilized targeted metabolomics to provide initial mechanistic insights into LEU nutritional intervention, it should be noted that this analysis—based on human biofluids—remains fundamentally exploratory at a phenotypic or correlative level when compared to controlled animal or cellular experiments. Although it can identify potential alterations in metabolic pathways, it cannot directly elucidate specific molecular targets or establish causal relationships.

## 5. Conclusions

In conclusion, a six-week intervention of leucine supplementation is associated with improved ankle muscle strength in endurance athletes. This effect may be related to modulation of BCAA metabolism.

## Figures and Tables

**Table 1:** Participant characteristics (Mean ± SD).

Characteristic	PLA (*n* = 10)	LEU (*n* = 10)	*p*
Age	16.55	16.76	0.76
Height (cm)	173.5	174.89	0.72
Body mass (kg)	61.82 ± 5.61	64.97 ± 8.36	0.06
BMI (kg/m^2^)	20.09 ± 1.24	21.10 ± 1.68	0.06
Fat mass (kg)	6.62 ± 1.91	7.97 ± 2.75	0.09
Body fat (%)	10.63 ± 2.74	11.97 ± 2.85	0.61
VO_2_max (L/min)	3.48 ± 0.49	3.60 ± 0.87	0.73

Note: BMI: body mass index; PLA: Placebo group; LEU: Leucine group; VO_2_max: Maximal Oxygen Uptake.

**Table 2:** Weekly training tracking data (Mean ± SD).

Index	Group	Week1	Week2	Week3	Week4	Week5	Week6
Training effect: Aerobic (0.0–5.0)	PLA	3.24 ± 0.84	3.8 ± 0.82	3.04 ± 1.25	3.37 ± 1.04	2.71 ± 1.3	2.94 ± 1.22
	LEU	3.18 ± 0.89	3.69 ± 0.88	3.1 ± 1.06	3.13 ± 1.04	2.65 ± 1.07	3.12 ± 1.21
	*p*	0.78	0.60	0.81	0.36	0.87	0.63
Training effect: Anaerobic (0.0–5.0)	PLA	1.8 ± 1.05	1.98 ± 0.93	1.78 ± 0.99	1.92 ± 1.23	1.29 ± 1.25	1.48 ± 1.2
	LEU	1.89 ± 0.96	1.97 ± 0.95	2.04 ± 0.77	1.75 ± 1.22	1.6 ± 1.03	1.63 ± 1.11
	*p*	0.70	0.96	0.21	0.56	0.40	0.66
TRIMP	PLA	216.55 ± 103.25	277.53 ± 91.36	241.52 ± 140.34	232.74 ± 110	174.15 ± 112.52	198.89 ± 110.09
	LEU	206.48 ± 105.19	254.64 ± 103.72	222.32 ± 127.9	208.42 ± 111.28	168.43 ± 103.2	209.97 ± 98.61
	*p*	0.65	0.33	0.53	0.38	0.87	0.73
Energy Expenditure (kcal)	PLA	1597.74 ± 677.03	1863.13 ± 355.4	1739.76 ± 657.16	1593.91 ± 602.77	1135.95 ± 668.17	1156.01 ± 469.59
	LEU	1606.62 ± 773.79	1825.86 ± 620.55	1651.61 ± 753.72	1359.45 ± 539.4	1248.2 ± 630.3	1348.56 ± 529.62
	*p*	0.95	0.76	0.59	0.10	0.59	0.22

Note: TRIMP: training impulse; PLA: Placebo group; LEU: Leucine group. The data presented in the table reflects the average values for training days each week (with the first six days designated as training days).

**Table 3:** Muscle strength (Mean ± SD).

	Index		PLA			LEU			*p* (6-Week (LEU/PLA))
			0-Week	6-Week	*p*	0-Week	6-Week	*p*	
	Vertical Jump (m)		0.36 ± 0.09	0.43 ± 0.10	0.17	0.32 ± 0.06	0.43 ± 0.09 **	0.01	0.86
60°	Ankle	P peak torque	56.89 ± 10.62	67.11 ± 12.63	0.08	44.00 ± 16.78	56.20 ± 14.04 ^#^	0.09	0.01
		D peak torque	159.67 ± 57.69	185.56 ± 62.04	0.37	167.30 ± 59.24	189.20 ± 45.01	0.36	0.76
	Knee	F peak torque	185.00 ± 45.01	178.00 ± 43.30	0.74	183.50 ± 62.36	185.10 ± 53.72	0.95	0.87
		E peak torque	316.78 ± 63.91	292.67 ± 60.91	0.43	304.70 ± 89.70	300.10 ± 75.09	0.90	0.92
180°	Ankle	P peak torque	40.44 ± 7.91	43.33 ± 5.24	0.38	31.50 ± 9.36	43.40 ± 10.06 *	0.01	0.12
		D peak torque	120.11 ± 27.90	133.00 ± 27.31	0.34	119.40 ± 40.00	129.40 ± 27.21	0.52	0.83
	Knee	F peak torque	158.22 ± 51.37	148.89 ± 39.48	0.67	153.10 ± 40.64	154.80 ± 31.55	0.92	0.98
		E peak torque	258.78 ± 67.88	251.89 ± 62.02	0.83	250.30 ± 58.96	255.30 ± 55.44	0.85	0.90

Note: The data presented in the isokinetic peak torque section were derived by calculating the sum of the peak torque values from both lower limbs. P: plantarflexion; D: dorsiflexion; F: flexion; E: extension; Group comparison: ^#^ *p* < 0.05; Within-group comparison: * *p* < 0.05, ** *p* < 0.01.

**Table 4:** Serum amino acid levels (μg/mL) (Mean ± SD).

Index	PLA			LEU			*p* (6-Week (LEU/PLA))
	0-Week	6-Week	*p*	0-Week	6-Week	*p*	
Leucine	13.29 ± 0.81	17.18 ± 0.73 **	0.00	14.20 ± 0.57	17.01 ± 0.81 **	0.00	0.75
Isoleucine	11.38 ± 0.44	28.16 ± 5.15 **	0.00	12.48 ± 0.41 **	26.50 ± 3.83 **	0.00	0.59
Valine	30.45 ± 0.65	36.7 ± 1.34 **	0.00	32.40 ± 1.70	39.30 ± 1.68 ^**#^	0.00	0.03

Note: Group comparison: ^#^ *p* < 0.05; Within-group comparison: ** *p* < 0.01.

**Table 5:** Differential metabolites between the LEU and PLA group.

	Name	Class	Fold Change	*p*	VIP
Up-regulated metabolites	β-Hyodeoxycholic acid	Bile acids	4.06	<0.001	2.79
	23-Norcholic acid	Bile acids	3.30	<0.001	2.73
	α-ketoisovaleric acid	Organic acids	1.41	0.03	1.38
	Glycylleucine	Amino acids and Peptides	1.32	0.03	1.33
Down-regulated metabolites	Desaminotyrosine	Phenylpropanoids and polyketides	0.57	0.03	1.77
	β-(m-Hydroxyphenyl) hydracrylic acid	Phenylpropanoids and polyketides	0.55	0.01	1.86

**Table 6:** Body composition (Mean ± SD).

Index	PLA			LEU			*p* (6-Week (LEU/PLA))
	0-Week	6-Week	*p*	0-Week	6-Week	*p*	
Body mass (kg)	61.82 ± 5.61	61.93 ± 5.42	0.97	64.97 ± 8.36	63.73 ± 7.18	0.73	0.55
BMI (kg/m^2^)	20.09 ± 1.24	20.12 ± 1.22	0.96	21.10 ± 1.68	21.13 ± 1.57	0.97	0.14
Fat mass (kg)	6.62 ± 1.91	6.21 ± 2.14	0.67	7.97 ± 2.75	8.10 ± 2.84	0.92	0.12
Body fat (%)	10.63 ± 2.74	9.91 ± 3.10	0.61	11.97 ± 2.85	12.19 ± 3.13	0.87	0.13

Note: BMI: body mass index.

**Table 7:** Aerobic capacity indicators (Mean ± SD).

Index	PLA			LEU			*p* (6-Week (LEU/PLA))
	0-Week	6-Week	*p*	0-Week	6-Week	*p*	
RER	1.10 ± 0.05	1.15 ± 0.05	0.05	1.05 ± 0.09	1.14 ± 0.05 *	0.02	0.57
VO_2_max (L/min)	3.48 ± 0.49	3.47 ± 0.53	0.52	3.60 ± 0.87	4.19 ± 0.54 ^#^	0.08	0.01

Note: RER: Respiratory exchange ratio; VO_2_max: Maximal oxygen consumption; Group comparison: ^#^ *p* < 0.05; Within-group comparison: * *p* < 0.05.

**Table 8:** Central fatigue indicators (Mean ± SD).

Index	PLA			LEU			*p* (6-Week (LEU/PLA))
	0-Week	6-Week	*p*	0-Week	6-Week	*p*	
0-RPE	14.33 ± 1.12	14.43 ± 1.90	0.74	14.70 ± 1.77	15.50 ± 1.78	0.33	0.25
5-RPE	10.89 ± 0.60	11.71 ± 2.06	0.41	11.00 ± 1.25	11.20 ± 1.32	0.73	0.54
10-RPE	8.78 ± 0.83	9.143 ± 2.19	0.76	8.8 ± 0.92	8.40 ± 1.075	0.38	0.37
Correct Rejection S	2.02 ± 0.54	1.87 ± 0.44	0.64	1.80 ± 0.18	1.68 ± 0.15	0.18	0.27
Correct Rejections T	113.83 ± 2.64	114.33 ± 2.66	0.75	114.25 ± 3.73	115.13 ± 2.85	0.61	0.61
Hit S	1.95 ± 0.70	1.86 ± 0.67	0.84	1.63 ± 0.17	1.58 ± 0.13	0.57	0.27
Hits T	73.67 ± 2.88	73.00 ± 2.83	0.69	74.25 ± 4.46	76.50 ± 1.93 ^#^	0.21	0.02
Hits PR	31.00 ± 25.04	25.17 ± 17.13	0.65	42.38 ± 35.17	57.00 ± 21.90 ^#^	0.34	0.01

Note: 0-RPE: Post-exercise immediate rating of perceived exertion; 5-RPE: Post-exercise 5-min rating of perceived exertion; 10-RPE: Post-exercise 10-min rating of perceived exertion; Correct Rejection S: Mean Correct Rejection Time (in seconds); Correct Rejections T: Total Correct Rejections; Hit S: Mean Hit Time (in seconds); Hits T: Total Hits; Hits PR: Percentile Rank of Hits. Group comparison: ^#^ *p* < 0.05.

## Data Availability

Data are available on request due to privacy and ethical restrictions.

## Supplementary Materials

The following supporting information can be downloaded at: https://www.mdpi.com/article/10.3390/metabo16020094/s1, Table S1: CONSORT 2025 checklist item description [49].

## Abbreviations

The following abbreviations are used in this manuscript:

LEU	Leucine
BCAAs	Branched-Chain Amino Acids
TE	Training Effects
TRIMP	Training IMPulse
HRR	Heart Rate Reserve
VO2max	Maximal Oxygen Consumption
RER	Respiratory Exchange Ratio
RPE	Rating of Perceived Exertion
Rejection S	Mean Correct Rejection Time
Hit S	Mean Hit Time
Rejections T	Total Correct Rejections
Hits T	Total Hits
Hits PR	Percentile Rank of HIts
LA	Lactic Acid
TC	Total Cholesterol
TG	Triglycerides
HDL-C	High-Density Lipoprotein Cholesterol
LDL-C	Low-Density Lipoprotein Cholesterol
CK	Creatine Kinase

## Appendix A

### Appendix A.1. Blood Biochemical Indicators Related to Lipid Metabolism

**Table A1:** Blood biochemical indicators related to lipid metabolism (Mean ± SD).

Index	PLA			LEU			*p* (6-Week (LEU/PLA))
	Before	After	*p*	Before	After	*p*	
TC (mmol/L)	3.89 ± 0.45	3.84 ± 0.23	0.78	3.93 ± 0.49	3.87 ± 0.43	0.80	0.84
TG (mmol/L)	0.75 ± 0.38	1.01 ± 0.65	0.32	0.68 ± 0.22	0.74 ± 0.12	0.40	0.22
HDL-C (mmol/L)	1.422 ± 0.17	1.30 ± 0.13	0.11	1.47 ± 0.28	1.41 ± 0.26	0.62	0.27
LDL-C (mmol/L)	2.00 ± 0.30	1.93 ± 0.27	0.62	1.98 ± 0.44	1.85 ± 0.35	0.46	0.57
VLDL-C (mmol/L)	0.34 ± 0.17	0.46 ± 0.30	0.32	0.31 ± 0.10	0.34 ± 0.05	0.40	0.22

Note: TC: Total Cholesterol; TG: Triglycerides; HDL-C: High-density lipoprotein cholesterol; LDL-C: Low-density lipoprotein cholesterol; VLDL-C: Very low-density lipoprotein Cholesterol.

### Appendix A.2. Hematological–Biochemical Indicators

The pre- and post-intervention data for the two groups are presented in Table A2. Compared to pre-intervention levels, CK and UA were significantly reduced in both the PLA and LEU groups after 6 weeks (*p* < 0.05), while levels of C and BUN were significantly increased (*p* < 0.05). Additionally, though not significant, the T levels in the PLA group as well as the LEU group were higher at the post-intervention test compared to pre-intervention (*p* > 0.05). However, when compared to the PLA group, no significant changes were observed in the relevant indicators in the LEU group after 6 weeks of intervention. Regarding the lactate acid level, LEU intervention significantly reduced post-exercise immediate lactate acid measure compared to PLA only at the week 0 following an acute exercise testing.

**Table A2:** Hematological–biochemical indicators (Mean ± SD).

Index	PLA			LEU			*p* (6-Week (LEU/PLA))
	0-Week	6-Week	*p*	0-Week	6-Week	*p*	
0-LA (mmol/L)	13.68 ± 2.62	14.96 ± 5.40	0.78	9.93 ± 2.60 ^#^	13.60 ± 4.50 *	0.04	0.58
5-LA (mmol/L)	12.02 ± 5.02	9.34 ± 2.91	0.08	9.92 ± 4.03	11.40 ± 3.54	0.39	0.23
10-LA (mmol/L)	10.03 ± 5.64	8.40 ± 3.90	0.31	8.82 ± 4.19	8.46 ± 1.43	0.80	0.97
CK (U/L)	807.67 ± 726.77	148.22 ± 25.62 *	0.03	734.10 ± 474.28	238.40 ± 125.63 **	0.01	0.05
C (µg/dL)	14.82 ± 2.85	18.09 ± 3.18 *	0.04	12.92 ± 2.60	18.94 ± 4.09 **	0.00	0.62
T (ng/dL)	468.63 ± 141.42	570.16 ± 105.94	0.10	479.78 ± 134.69	521.21 ± 103.05	0.45	0.32
T/C	33.51 ± 14.64	32.14 ± 7.78	0.81	38.21 ± 11.55	28.92 ± 8.67	0.06	0.41
UA (µmol/L)	414.31 ± 95.68	318.62 ± 35.08 *	0.01	442.62 ± 78.91	346.33 ± 68.90 **	0.01	0.29
Cr (µmol/L)	67.17 ± 10.23	76.11 ± 10.72	0.09	67.81 ± 8.42	76.41 ± 10.87	0.06	0.95
BUN (mmol/L)	4.98 ± 1.35	6.07 ± 1.08	0.08	5.12 ± 0.89	6.05 ± 0.80 *	0.03	0.96

Note: 0-LA: Post-exercise immediate lactate; 5-LA: Post-exercise 5-min lactate; 10-LA: Post-exercise 10-min lactate; CK: Creatine Kinase; C: Cortisol; T: Testosterone; UA: Uric acid; Cr: Creatinine; BUN: Blood urea nitrogen; Group comparison: ^#^ *p* < 0.05; Within-group comparison: * *p* < 0.05, ** *p* < 0.01.

### Appendix A.3. Serum Antioxidant INDICATORS

As shown in Table A3, compared to pre-intervention levels, GSH and TBIL were significantly reduced in both the PLA and LEU groups after 6 weeks (*p* < 0.05), while MDA levels in the PLA group were significantly increased compared to pre-intervention levels (*p* < 0.05). However, no significant changes in MDA were observed in the LEU group after 6 weeks of intervention, as well as no significant changes in MDA when compared to the PLA group. SOD was significantly down-regulated only in LEU group (*p* < 0.05).

**Table A3:** Serum antioxidant indicators (Mean ± SD).

Index	PLA			LEU			*p* (6-Week (LEU/PLA))
	0-Week	6-Week	*p*	0-Week	6-Week	*p*	
GSH (µmol/L)	6.75 ± 3.15	1.99 ± 0.94 **	0.00	6.91 ± 2.35	3.11 ± 1.94 **	0.00	0.13
MDA (nmol/mL)	13.70 ± 8.33	27.19 ± 10.34 *	0.01	21.80 ± 20.74	24.80 ± 13.75	0.71	0.68
SOD (U/mL)	68.90 ± 1.69	62.67 ± 8.45	0.06	67.65 ± 3.27	63.42 ± 4.24 *	0.02	0.81
TBIL (µmol/L)	23.99 ± 8.31	15.23 ± 5.60 *	0.02	24.66 ± 11.05	16.37 ± 3.57 *	0.05	0.60

Note: GSH: Glutathione; MDA: Malondialdehyde; SOD: Superoxide Dismutase; TBIL: Total Bilirubin; Within-group comparison: * *p* < 0.05, ** *p* < 0.01.

### Appendix A.4. Serum Amino Acid Levels

**Table A4:** Serum amino acid levels (μg/mL) (Mean ± SD).

Index	PLA			LEU			*p* (6-Week (LEU/PLA))
	0-Week	6-Week	*p*	0-Week	6-Week	*p*	
Citrulline	4.87 ± 0.63	5.33 ± 0.57	0.31	4.96 ± 0.31	5.39 ± 0.44	0.10	0.85
Glutamic acid	12.62 ± 1.02	12.48 ± 0.64	0.81	12.83 ± 0.68	12.92 ± 0.64	0.84	0.33
Glutamine	76.48 ± 6.02	94.38 ± 4.28 **	0.00	77.19 ± 5.39	89.11 ± 3.44 **	0.00	0.07
Histidine	14.71 ± 1.17	15.30 ± 0.42	0.39	14.45 ± 0.78	14.87 ± 0.26	0.29	0.09
Leucine	13.29 ± 0.81	17.18 ± 0.73 **	0.00	14.20 ± 0.57	17.01 ± 0.81 **	0.00	0.75
Isoleucine	11.38 ± 0.44	28.16 ± 5.15 **	0.00	12.48 ± 0.41 **	26.50 ± 3.83 **	0.00	0.59
Lysine	26.43 ± 1.90	28.06 ± 2.09	0.28	25.40 ± 0.97	27.89 ± 2.32	0.06	0.91
Methionine	5.06 ± 0.41	5.61 ± 0.30	0.06	4.95 ± 0.48	5.36 ± 0.55	0.24	0.44
Ornithine	10.78 ± 0.82	12.58 ± 0.89 *	0.02	10.26 ± 0.86	11.80 ± 0.58 **	0.00	0.15
Phenylalanine	13.03 ± 0.64	17.46 ± 0.74 **	0.00	13.06 ± 0.88	17.75 ± 0.52 **	0.00	0.51
Proline	18.94 ± 4.24	8.89 ± 2.95 *	0.01	20.38 ± 2.06	8.96 ± 1.73 **	0.00	0.96
Serine	17.37 ± 1.82	19.37 ± 0.44	0.09	16.55 ± 0.91	18.33 ± 1.24 *	0.03	0.14
Tryptophan	11.62 ± 0.50	17.20 ± 0.87 **	0.00	12.69 ± 0.43 **	17.32 ± 0.67 **	0.00	0.82
Tyrosine	10.42 ± 0.56	13.51 ± 0.62 **	0.00	10.62 ± 0.99	13.36 ± 0.87 **	0.00	0.78
Valine	30.45 ± 0.65	36.70 ± 1.34 **	0.00	32.40 ± 1.70	39.30 ± 1.68 ^**#^	0.00	0.03
Arginine	16.13 ± 2.31	23.87 ± 2.67 **	0.00	16.27 ± 2.04	21.95 ± 1.81 **	0.00	0.10
Alanine	36.97 ± 5.33	46.69 ± 7.68 *	0.01	37.01 ± 7.39	43.09 ± 7.41	0.10	0.34
Glycine	29.99 ± 5.17	39.86 ± 4.65 **	0.00	27.70 ± 4.31	36.71 ± 3.04 **	0.00	0.11
Threonine	15.09 ± 1.56	21.33 ± 1.79 **	0.00	14.17 ± 2.22	18.95 ± 3.40 **	0.00	0.09
Asparagine	6.82 ± 0.60	7.17 ± 0.23	0.33	6.07 ± 0.66	6.65 ± 0.51	0.15	0.09
Aspartic acid	3.15 ± 0.26	4.96 ± 0.57 **	0.00	3.29 ± 0.31	4.93 ± 0.32 **	0.00	0.94

Note: Group comparison: ^#^ *p* < 0.05; Within-group comparison: * *p* < 0.05, ** *p* < 0.01.

### Appendix A.5. Correlation Analysis Between Differential Metabolites and Routine Indicators

**Table A5:** The correlation of differential metabolites and exercise performance indicators (R value).

Group	Name	Vertical Jump	60° Ankle Plantar Peak Torque	60° Ankle Dorsiflex Peak Torque	VO_2_max	Hits T	Hits TR
PLA (6-week/0-week)	Glycodeoxycholic acid	0.33	0.29	0.41	−0.50	0.36	0.37
	Glycochenodeoxycholic acid	0.19	0.22	0.48 *	−0.58 *	0.38	0.40
	Murocholic acid	0.18	−0.01	−0.00	−0.16	0.13	0.11
	Choleic Acid	0.17	−0.01	−0.01	−0.15	0.12	0.11
	Deoxycholic acid	0.21	−0.02	0.07	−0.20	0.16	0.13
	Undecylenic acid	0.02	−0.50 *	−0.06	0.77 **	−0.38	−0.33
	Phthalic acid	−0.31	−0.52 *	−0.28	0.73 **	−0.39	−0.36
	Dodecanoic acid	−0.17	−0.46	−0.16	0.30	−0.54 *	−0.46
	Diethyl oxalpropionate	−0.10	−0.53 *	−0.11	0.33	−0.56 *	−0.46
	β-Alanine	−0.25	−0.49 *	−0.33	0.70 **	−0.49	−0.48
LEU (6-week/0-week)	5β-CHOLANIC ACID METHYL ESTER	0.55 *	0.21	0.26	−0.42	−0.66 *	−0.48
	Glycochenodeoxycholic acid	0.39	0.51 *	0.23	−0.25	0.15	0.07
	Glycocholic acid	0.49 *	0.61 **	0.30	−0.25	0.04	0.00
	Citramalic acid	0.29	0.38	0.38	−0.43	−0.05	−0.01
	Retinal	0.34	0.49 *	0.39	−0.55 *	0.03	−0.09
	Sebacic acid	−0.37	−0.38	−0.06	0.29	0.10	0.05
	3,6-Diketocholanic Acid Methyl Ester	−0.38	−0.50 *	−0.12	0.36	−0.21	−0.29
	Guanosine monophosphate	−0.32	−0.30	−0.38	0.13	0.37	0.45
	β-Alanine	−0.61 **	−0.55 *	−0.41	0.60 **	0.13	0.13
	Phthalic acid	−0.58 **	−0.51 *	−0.27	0.51 *	0.13	0.12
6 week (LEU/PLA)	β-Hyodeoxycholic Acid	−0.22	−0.41	−0.07	−0.12	−0.25	−0.27
	23-Norcholic Acid	−0.23	−0.42	−0.03	−0.05	−0.22	−0.22
	α-ketoisovaleric acid	−0.38	−0.44	−0.09	0.42	0.11	0.06
	Glycylleucine	−0.18	0.09	0.30	0.07	−0.09	−0.10
	Desaminotyrosine	0.03	0.21	0.03	0.09	0.01	−0.09
	β-(m-Hydroxyphenyl)hydracrylic Acid	0.21	0.06	−0.03	−0.03	0.34	0.34

Note: PLA: Placebo group; LEU: Leucine group; Within-group comparison: * *p* < 0.05, ** *p* < 0.01.
